# Normalization and Gene p-Value Estimation: Issues in Microarray Data Processing

**DOI:** 10.4137/bbi.s441

**Published:** 2008-05-28

**Authors:** Katrin Fundel, Robert Küffner, Thomas Aigner, Ralf Zimmer

**Affiliations:** 1 Institut für Informatik, Ludwig-Maximilians-Universität München, Amalienstrasse 17, 80333 München, Germany; 2 Institut für Pathologie, Universitätsklinikum Leipzig, Liebigstr. 26, 04103 Leipzig, Germany

**Keywords:** expression data, normalization, regulated genes, data processing

## Abstract

**Introduction:**

Numerous methods exist for basic processing, e.g. normalization, of microarray gene expression data. These methods have an important effect on the final analysis outcome. Therefore, it is crucial to select methods appropriate for a given dataset in order to assure the validity and reliability of expression data analysis.

Furthermore, biological interpretation requires expression values for genes, which are often represented by several spots or probe sets on a microarray. How to best integrate spot/probe set values into gene values has so far been a somewhat neglected problem.

**Results:**

We present a case study comparing different between-array normalization methods with respect to the identification of differentially expressed genes. Our results show that it is feasible and necessary to use prior knowledge on gene expression measurements to select an adequate normalization method for the given data. Furthermore, we provide evidence that combining spot/probe set p-values into gene p-values for detecting differentially expressed genes has advantages compared to combining expression values for spots/probe sets into gene expression values. The comparison of different methods suggests to use Stouffer’s method for this purpose.

The study has been conducted on gene expression experiments investigating human joint cartilage samples of Osteoarthritis related groups: a cDNA microarray (83 samples, four groups) and an Affymetrix (26 samples, two groups) data set.

**Conclusion:**

The apparently straight forward steps of gene expression data analysis, e.g. between-array normalization and detection of differentially regulated genes, can be accomplished by numerous different methods. We analyzed multiple methods and the possible effects and thereby demonstrate the importance of the single decisions taken during data processing. We give guidelines for evaluating normalization outcomes. An overview of these effects via appropriate measures and plots compared to prior knowledge is essential for the biological interpretation of gene expression measurements.

## Introduction

Today, numerous methods and tools exist for analyzing gene expression data. Many normalization techniques exist, e.g. [1,2,3,4], as well as methods for detecting differentially expressed genes, e.g. [5,6,7,8,9]. Specific databases for gene expression data have been set up (e.g. [10]), and software packages have been developed for analyzing microarray data in a largely automated way, e.g. [11,12,13,14,15], many of them integrate gene expression data with further information obtained from e.g. ontologies, pathway databases or text mining.

It is known that the ‘higher-level’ outcome, e.g. a list of differentially regulated genes, of any micro-array experiment depends on the ‘low-level’ details of data processing. One important step in data processing is normalization. Yet, comparisons of normalization methods presented so far [16,17] do not consider sample groups. Generally, existing literature offers little guidance on how to decide which method to use, how to compare different methods and their outcomes, and how to check possible outcomes against biological expectation and downstream interpretation.

We focus on the analysis of between-array normalization steps, i.e. we assume the data to be analyzed has already passed a primary normalization covering for variances due to localization of the probe sequences on the array, GC-content of the probe sequences, varying sensitivity in different detection ranges, and others. Numerous methods are available for this primary normalization (e.g. the default Affymetrix method MAS 5.0, RMA [18], dCHIP [19], for an overview see [20]), these are not in the focus of the present study.

Furthermore, biomedical or biological interpretation of microarray experiments require p-values and fold-changes for genes rather than spots. Microarrays often contain several spots or probe sets that represent the same gene but are not necessarily identical. Typically they cover different sequence segments within the entire target sequence, or which sometimes represent splice variants. They vary in terms of binding affnity and specificity, and, consequently, yield varying intensities.

For interpretation and subsequent analysis, combination of spot information into gene information becomes essential. This represents a somewhat neglected problem. Generally, the average of the individual spot/probe set expression values is taken as gene expression level. If the expression intensities vary due to the biochemical and physical effects described above, the spot/probe set yielding the highest intensity has the most significant influence on the overall mean. Thus the spots or probe sets representing the same gene do not contribute equally to the overall result.

Individual spots representing the same gene can be seen as individual tests (independent to a certain degree) of the same null hypothesis. For interpretation, we aim at combining the results of these tests to ask whether there is evidence from the collection of tests that we might reject the null hypothesis of no differential expression. The collection of methods known as meta-analysis gives many ways to perform these combinations, including some techniques that combine p-values [21,22].

The combination of p-values represents a method to circumvent the combination of expression levels and thus to overcome the possible inconsistencies as it represents a scale-free method to combine spot information into gene information. For each spot or probe set, a p-value is calculated, and these spot p-values are subsequently combined into a gene p-value.

The focus of this study is twofold. The first is to compare a number of between-array normalization methods by analysis of their effects on the differentially expressed genes deduced from an exemplary data set for which we have some background knowledge. The large number of samples allows us to perform a stability analysis on the significantly regulated genes. Recently, it has been shown [23] that in numerous published large studies on gene expression differentially expressed genes are highly unstable for subsets of the analyzed samples. Thus, as a gold standard is not available, we propose a procedure which estimates the errors and quantifies their amount via a robustness analysis.

Secondly, we provide evidence that combining spot/probe set p-values into gene p-values has advantages compared to combining intensities. We compare different methods for combining spot p-values into gene p-values and suggest a method for this task.

We present a case study conducted on two gene expression data sets of human joint cartilage samples. The focus is on a large data set representing 83 samples classified into four disease-related groups of osteoarthritis (OA). For reviews on osteoarthritis see [24,25,26]. The data was collected to identify genes differentially regulated between pairs of sample groups as these genes are of potential interest for understanding disease mechanisms, for diagnosis and medical therapy. cDNA arrays allowed for analysis of the four distinct groups as they require smaller amounts of sample material. The large number of samples allows us to perform a robustness analysis of derived p-values. Furthermore, we analyzed a data set obtained with Affymetrix GeneChips investigating 26 samples of human joint cartilage classified into two disease-related groups. Importantly, these data sets allowed us to make use of existing background knowledge on the disease progress and specificities concerning individual disease stages.

We focus on data processing effects that need to be considered not only in the case of cDNA arrays but also for other types of arrays (here only shown exemplarily for Affymetrix GeneChips). Although structure and distribution of the data is somewhat different, similar effects can be observed and similar measures can be applied. Regardless of the chip technology in-between array normalization is required and probe sets representing the same gene show significant variances in fold-changes and p-values. Special care is necessary to deal with probe sets representing the same gene that show opposite regulation in a number of cases.

## Methods

### Dataset

The data analyzed in the present study was obtained from a custom designed cDNA microarray. The experiment, including array production, hybridization, scanning by phosphorimaging, and primary data analysis (i.e. local background correction, removal of outlier spots, within array normalization, and expression value determination) was performed by GPC-Biotech AG (Martinsried, Germany). A part of the spotted cDNA species had been preselected for OA-relevant genes. Scanning was done by phosphorimaging. Each microarray contains 7467 spots, 5517 spots represent 3648 genes, there are 1 to 74 spots per gene on the array, and 1062 genes are represented by more than one spot.

The data set is described in the following as: *X* = *X_ks_* = {*x_ks_*|*k* = 1 … 83, *s* = 1 … 7467}, where *s*: spots, *k*: samples.

**Figure 1:**
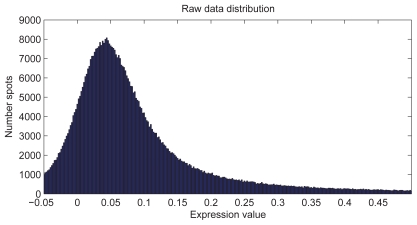
Raw data distribution: Distribution of raw data of the analyzed dataset (before outlier removal, i.e. 83*7467 spots). Background-correction lead to negative values.

83 samples of human joint cartilage were analyzed, 5 of them were outlier and thus removed (details not shown here). The raw data of the remaining 78 samples represents the starting point for all further analysis. The expression value distribution is given in Figure 1.

The samples were classified based on histological criteria: 18 normal (*n*), 20 early degenerative cartilage (*e*), 21 peripheral OA (*p*), and 19 central OA (*c*). The class ‘late OA’ (*l*) was defined as the combined set of peripheral and central OA, this represents all samples of patients severely affected by Osteoarthritis.

One of the main goals of the experiment was to identify differentially regulated genes for the group pairs *ne, np, nc, ep, ec, pc, nl, el*. For the subsequent biomedical analysis, genes differentially expressed between specific pairs of disease stages (especially *ne, nl,* and *pc*) are of primary interest as these provide specific information on the individual steps of disease progress, and thus on the individual disease stages.

Given the diffculty of obtaining human joint samples this represents a large data set. The group sizes of about 20 samples allow for statistical robustness and quality analysis. The used cDNA microarrays require significantly smaller amounts of sample material than e.g. Affymetrix arrays and thus make it possible to measure gene expression for samples that contain very low amounts of RNA like the disease stage ‘central’ that is characterized by nearly complete cartilage loss. The full dataset used for this study and its biological interpretation has been published separately [27].

The Affymetrix data set contains data from 26 U133 Plus 2.0 arrays measuring healthy and osteo-arthritic cartilage (13 samples each). This array contains 1–20 probe sets per Entrez Gene identifier (manuscript in preparation).

#### Background knowledge

For the given data, the following background knowledge was available and corresponding expectations apply for data processing:

1. It was experimentally confirmed that mRNA content was the same for all sample preparations, and thus expression intensities are expected to be similar for all measurements.
2. The number of up- and downregulated genes is expected to be balanced for each comparison. This expectation applies to many experiments, exceptions encompass e.g. stimulation experiments.
3. From previous experiments, it is known that the degree of similarity varies substantially between the sample classes. Specifically, *n* and *e* as well as *p* and *c* are very similar, whereas *n* is very different from *p* and *c* and, consequently, also from *l*. Previous cluster analysis showed a good separation between the class pairs *ne* and *pc*, whereas the groups *n* and *e* as well as *p* and *c* were not separated from each other (results not shown here). Interestingly, also in terms of clinical staging, *n* and *e* and *p* and *c* resemble each other, whereas the two group pairs *ne/pc* are clearly distinct. The result of the cluster analysis lead to the expectation that more genes are significantly regulated in the comparisons *np*, *nc*, and *nl* than in the comparisons *ne* and *pc*.

In the following we briefly describe the methods applied for data processing and procedures for control of the biological expectations.

### Normalization

We applied the following standard normalization methods (For an overview see [28,29,30], for details see indicated references):

*Globalization* assumes that the overall mRNA content of each sample and thus the total intensity is the same for each array. Normalization is achieved via dividing intensities by the total intensity of the given array.

*Centralization* [31] is a normalization method that estimates for each pair of arrays the factor of proportionality. From the resulting matrix of pairwise factors, an optimally consistent scaling is determined. This results in a multiplicative factor for each array. Centralization requires as parameter the range of reliable measurements; for the given data we estimated 0.03–1.

*Percentile Normalization* is a method that adjusts a certain selected percentile to the same level for all samples by applying a multiplicative factor to each sample. We used the 50% (eq. global median location normalization) and 75% percentiles, which are typically used.

*MAD Scale Normalization* [32,33] adjusts the median and MAD (median absolute deviation), which are robust measures for the location and spread of distributions, of all arrays to a common level. The median and MAD of each array are set to the respective measure of the entire data set. For each sample *k* and spot *s* the original value *x_ks_* is transformed into the normalized value *x_ks_*^″^ according to the following equation:

$$x_{ks}^{\prime \prime }=\frac{x_{ks}-\text{median}({\text{x}}_{\text{k}})}{\text{MAD}({\text{x}}_{\text{k}})}$$

$$\begin{array}{c} x_{ks}^{\prime \prime }=x_{ks}^{\prime }*\text{MAD}(\text{X})+\text{median}(\text{X})\\ \text{MAD}({\text{x}}_{\text{k}})=\text{median}(|{\text{x}}_{\text{k}}-\text{median}({\text{x}}_{\text{k}}\cdot )|) \end{array}$$

where: *MAD*: median absolute deviation; *k*: sample; *s*: spot measured in sample *k; X*: entire dataset.

*Variance Stabilization* [34] incorporates data calibration, an intensity-dependent error model and data transformation; it is intended to lead to a measure of differential expression which is independent of the mean intensity.

*LOESS* [35] (Local Regression) fits simple models to subsets of the data; thus it does not require to specify a global function of any form to fit a model to the data, only to fit segments of the data, where segments are defined by measured intensity.

*Quantile Normalization* normalizes the distributions of the expression values, i.e. each quantile, for each array.

#### Flooring

The background level was estimated to be at 0.01 (details not shown here) and expression values <0.01 were set to 0.01 for all further analysis. Overall, approx. 17% of the expression values were set to the floor threshold value; spots assigned with the floor threshold for all samples are not considered for further analysis (26 spots).

### Differential expression

#### p-value combination

Differently expressed genes were detected based on the following procedure: First, the two-sided Wilcoxon ranksum test was applied for calculation of p-values for spots. Next, these spot p-values are combined to obtain overall **gene p-values**. We applied three different methods for combining spot p-values into gene p-values:

1. **Fisher’s inverse chi-square method** [36]. This method uses the fact that given a uniform distribution *U*, −2 * *log*(*U*) has a chi-square distribution with two degrees of freedom, and the sum of two independent chi-square variables is again chi-square distributed (with four degrees of freedom). Consequently, the combined p-value *p_chi_*(*g*) for a gene *g* can be computed as:
  $$p_{chi}(g)=1-X_{2d}^{2}\left(\sum_{s}-2*log(ps)\right)$$
  where *p_s_* are the p-values for spots s representing gene *g* (in our case obtained from the two-sided Wilcoxon ranksum test), *d* is the number of spots s representing gene *g,* and *X_d_*^2^(*x*) is the cumulative distribution function of the chi-square distribution with *d* degrees of freedom.
2. **A variant of Fisher’s inverse chi-square method** that also considers the directions associated to individual spot p-values:
  $$p_{dirchi}(g)=\text{min}\left(1-X_{2d}^{2}\left(\sum_{s}-2*log(p_{s}^{dir})\right)\right)$$
  where *p_s_^dir^* are the onesided spot p-values (Wilcoxon ranksum test) for all spots s representing gene g; these onesided spot p-values are determined for both regulation directions; the overall combined gene p-value then equals to the smaller of the two combined p-values, each of them corresponding to one test direction.
3. **Stouffer’s method** [37]. This method transforms p-values to z-scores assuming a normal distribution (*p*_s_ → *Z*_s_) by
  $$Z_{s}=\varphi^{-1}(1-p_{s}^{onesided})$$
  Each *Z_s_* gets the sign of the log_2_(fold change) of the corresponding spot. The z-scores of spots representing one gene are summed, and the sum is scaled:
  $$Z_{overall}=\sum_{s}Z_{s}/\sqrt{k}$$
  where *k* is the number of tests, i.e. the number of spots to be combined. Finally the z-scores are transformed back to p-values (Z*_overall_* → *p_overall_*) by
  $$p_{overall}^{onesided}=\frac{1}{\sqrt{2\pi }}\int_{-\text{inf}}^{-|{\text{Z}}_{overall}|}e^{\frac{-t^{2}}{2}}dt$$

##### Gene p-values obtained without p-value combination

Mean expression values over all spots representing a gene were calculated for each sample and gene, and subsequently Wilcoxon ranksum p-values from these mean expression values were determined (mean expr. value). Given the individual spot p-values of the spots representing a gene, we used for each gene the most (min. p-value) and least (max. p-value) significant corresponding spot p-value as gene p-value.

#### Fold change

Given two sample groups *C_1_, C_2_* ∈ {*n, e, p, c, l*}, *C_1_* ≠ *C_2_* the overall fold-change for a gene *g* was estimated as follows: A spot s for the gene *g* is taken into account if at least one expression value in the groups under investigation is above the floor value (0.01); for each spot we compute fold-changes (sfc_sg_^*C*_1_,*C*_2_^) for all pairs of samples derived from the two groups to be compared. The median of these spot fold-changes is used as overall estimate for the gene-fold change (*fc*(*g*)^*C*_1_,*C*_2_^).

$$\begin{array}{c} S_{g}^{\prime }:=\{s\;spot|s\;\text{represents gene g}\}\\ s\in S_{g}:=\{s\in S_{g}^{\prime }|\exists k\in \{C_{1}\cup C_{2}\}:{\text{X}}_{\text{ks}}>0.01\}\\ {\text{sfc}}_{{\text{S}}_{\text{g}}}^{{\text{C}}_{1},{\text{C}}_{2}}:=\left\{{\text{log}}_{2}({\text{x}}_{\text{is}}/\text{exp}\;{\text{r}}_{\text{js}})|\text{i}\rceil {\text{C}}_{1}\wedge \text{j}\rceil {\text{C}}_{2},\text{s}\rceil {\text{S}}_{\text{g}}\right\}\\ \text{fc}{\left(\text{g}\right)}^{{\text{C}}_{1},{\text{C}}_{2}}=2^{\text{median}({\text{sfc}}_{{\text{S}}_{\text{g}}}^{{\text{C}}_{1},{\text{C}}_{2}})} \end{array}$$

where: x_ks_ is the expression value of spot *s* in sample *k*.

We apply different methods for combining spot p-values into gene p-values, and one method for computing fold changes. In rare cases, the independent determination of gene-fold change and directed gene p-value causes sign inconsistencies for gene p-value and fold change. We analyzed the data for this and found that this effect only occurs for few genes with fold changes that are very close to 1; therefore this does not imply problems for further biological interpretation of data.

### Number of regulated genes

The gene p-values are converted into q-values by use of the R-library ‘qvalue’[38]. The q-value quantifies the false discovery rate, its computation implies the estimation of the number of non-regulated genes π_0_ from the p-value distribution via bootstrapping. 1−π_0_ thus estimates the number of regulated genes, which we use as quality measure for evaluating appropriateness of normalization.

### Robustness analysis

#### Leave-one-out analysis

Leave-one-out analysis allows for estimating the robustness of p-value calculation, i.e. one sample is disregarded at a time and p-values are calculated based on the remaining samples. The resulting lists of p-values were compared to the list derived from the full dataset. This analysis was conducted with the Stouffer method for combining p-values.

For estimating the robustness, the p-values obtained from the full dataset were considered as standard of truth. A series of cutoff-p-values (between 10^−7^ and 10^−1^) is applied and for each of these, the fraction of significantly regulated genes from the full dataset that are also significantly regulated to the given cut-off p-value in the leave-one-out datasets is determined. We selected ‘robust’ differentially expressed genes according to two criteria:

- *exact*: The fraction of genes that are significant in all leave-one-out datasets.
- *relaxed*: The fraction of genes that are significant with a p-value of ≤2* the cutoff p-value in all leave-one-out datasets.

#### Subset sampling

The subset sampling analysis is used for estimating the robustness of the most significantly regulated genes for a given group comparison. For each group pair, 50 random sample subsets (*m* = 10 … 18 samples used for each of the groups to be compared) are generated and p-values are calculated based on these subsets. Next, the top p-value genes are analyzed; we used the *t* top genes obtained from the entire sample set as standard of truth and determined the fraction of these top candidates that are also among the *t* top candidates of at least *s*% of the subset p-value sets. For *t* we used 50, 75, 100; for s we used 100, 80, 50.

## Results and Discussion

In the following we describe the results of various methods for normalization and gene p-value determination; we focus on the analysis of plausibility criteria and the correspondence of outcomes to the available background knowledge described in the methods section (see Data—Background knowledge).

### Normalization

#### Effect on expression levels

**Figure 2:**
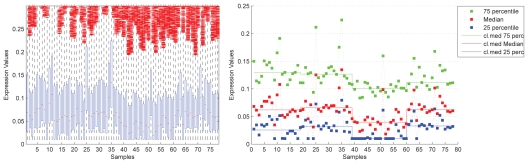
Boxplot and group-level plot: Boxplot (left panel) and group-level plot (right panel) for the same data. The boxplot shows the range between the 25% and 75% percentiles as blue bars and the median as red mark in the blue bar. The group-level plot shows the 25% (green), 50% (red) and 75% (blue) percentile for each sample (as does the boxplot) and additionally shows the median over these values for each sample group representing different disease stages (*n*: samples 1–18, *e*: 19–38, p: 39–59, c: 60–78; cl.med: class median).

The effect of normalization on data is typically evaluated by visual inspection of boxplots (Fig. 2, left panel). A boxplot shows the individual samples on the x-axis and the expression values on the y-axis. The 25% percentile and 75% percentile of a dataset are shown as lower and upper boundary of a box and the median as horizontal line within the box, the whisker length is typically proportional to the interquartile range, and all data points lying outside these whiskers are displayed individually as outliers. While boxplots are easy to generate and interpret, we suggest in addition to these a different type of plot for evaluating the effect of normalization, especially for experiments dealing with sample groups.

This **group-level plot** also shows the 25%, 50% and 75% percentiles for the individual samples as does the boxplot. Data displayed as outlier in box-plots is ignored as it is not in the focus of normalization. Most importantly the plot additionally shows the group-levels of the plotted percentiles, i.e. the median of the corresponding percentile over all samples belonging to the same group. This group-level allows to identify group-specific variations within data, which may not be inherent to the biological samples under investigation. Figure 2 shows a boxplot and group-level plot for our dataset. For the investigated samples, analysis of total mRNA content showed no group specific variations on the mRNA level; variations must be due to experimental setup or any other undesired effect. The group-level plot clearly shows the different levels of expression data for the different sample groups, in the boxplot this is much less evident.

#### Effect on differential expression

**Figure 3:**
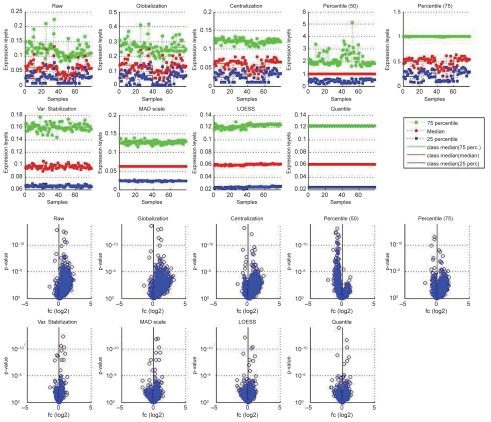
Effects of normalization on p-value and fold-change determination: group-level plots for all samples and volcano plots for group comparison *p* versus *c* (*p*: 39–59, *c*: 60–78) for raw data and different between-array normalizations (for details see section ‘Normalization’).

From prior knowledge we expect that up- and down-regulation events should be approximately balanced. If the group expression levels are significantly different we might not be able to observe the expected behavior. Figure 3 contrasts normalized data with raw data for the comparison *pc*, it shows group-level plots and the resulting p-values and fold changes. The raw and centralized data yield asymmetric fold-change distributions, more genes appear upregulated than downregulated from *p* to *c* due to the differences in group level. The 50% percentile normalization produces more downregulated than upregulated genes. Only a subset of the analyzed methods yield approximately symmetric distributions, these are the 75% percentile normalization, MAD scale normalization, Variance Stabilization, LOESS, and Quantile Normalization.

Between-array normalization aims at removing systematic effects occurring between arrays, e.g. when one array yields systematically higher expression values than a second one even though hybridized with the same sample. Various methods exist for between-array normalization, they vary significantly in how rigorously they modify the original data. Some of the methods apply a multiplicative factor that depends on the total of expression values of the considered array (e.g. globalization) or a single characteristic value of the distribution of expression values (e.g. percentile normalization), or on the expression values of the set of arrays under consideration (e.g. centralization). More stringent normalization methods apply a multiplicative and an additive factor, i.e. they modify location and spread of the original distribution (e.g. scale normalization). Finally, some normalization methods fit the location and the shape of the original distribution (e.g. quantile normalization), or adjust data in a intensity-dependent way (e.g. variance stabilization, LOESS). This class of methods clearly modifies the original data the most.

Generally, normalization is intended to modify the underlying data as slightly as possible, but as much as necessary to remove systematic biases yet to conserve biological relevant information. Far more normalization techniques than the ones analyzed here exist (e.g. [39,33,1,40]); most of the newer normalization techniques are non-linear as this is assumed to perform generally better than linear techniques. Some of them focus on two-channel or Affymetrix-type data and can therefore not easily be applied to other kind of data, others can directly or after slight adaptation be applied to e.g. one-channel cDNA microarray data. The study presented here concentrated on a number of normalization techniques and shows that different normalization methods yield different results, thus the normalization method should be selected in accordance to the data set under investigation with the general guideline ‘enough’ normalization with only slight data modification.

### Deriving p-values for genes

#### General considerations

Within microarrays, the number of spots/probe sets per gene typically varies. Different cDNAs representing the same gene do not need to be identical, they can represent splice variants or cover distinct regions within the gene sequence, which can result in high variability of measured expression intensities. In the case of oligonucleotide arrays, the individual oligos representing a gene vary in binding affnity (i.e. sensitivity) and specificity and the annotation of individual spots/probe sets varies in reliability. These issues complicate the interpretaion of expression data. On virtually all current microarray platforms, a subset of the genes are represented by multiple spots or probe sets. Generally, the decision how to deal with this multiplicity is deferred to the user. The individual spots or oligos thus can return varying expression values; these result from experimental methodology and should not always be considered as measurement errors. The naive combination of spot expression values into gene expression values can lead to biased results as spots or probe sets of varying intensity do not contribute equally. Another approach that is frequently taken is to take the most or least significant spot representing a gene. This yields the most optimistic or most conservative estimate of real significance, and consequently is very susceptible to outlier p-values.

We propose to determine gene p-values based on spot p-values. To our knowledge, gene p-value determination has not been addressed by spot p-value combination, and this is the only comprehensive analysis about how to best combine spot p-values into gene p-values available.

Unfortunately, large scale gold standards are not available. So correct fold-changes and significances for all genes represented by more than one spot/probe set on an array are not known. Some spike-in experiments are publicly available (e.g. [41,42]). These are useful for evaluating methods for the determination of expression values, but they make no assertions on differentially expressed genes represented by more than one spot/probe set. Confirmation of differential expression can be obtained from alternative techniques like quantitative PCR. We verified 10 genes by quantitative PCR [27] and the results showed good agreement with our proposed method.

#### Requirements

We propose a number of heuristic yet intuitive criteria for the evaluation of gene p-values:

- if all spots are regulated in the same direction, then the gene p-value should be at least as significant as the least significant spot p-value.
- if spots show inconsistent direction of regulation, then the gene p-value should be of lower significance than the most significant spot p-value.
- for spots showing inconsistent direction of regulation and of approximately equal significance the gene p-value should tend towards 1.

These criteria reflect our interest in differentially expressed genes. For studies with an alternative focus, such as detection of alternative splicing, other criteria could be set up.

#### Evaluation

**Figure 4:**
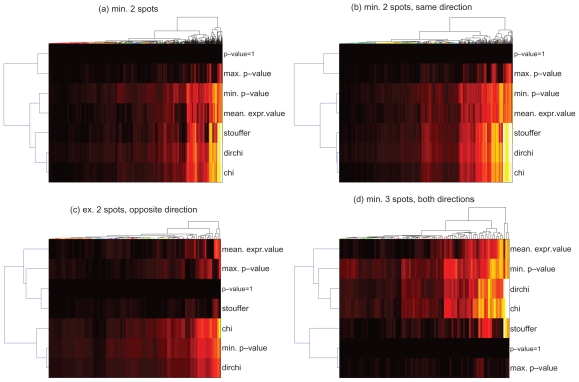
p-value combination: Comparison of different methods for combining spot p-values to gene p-values by their overall results. Plots show p-values (log10 transformed, black: least significant p-value, white: most significant p-value) for genes (columns) obtained from various combination methods (rows). (p-value = 1) is added as baseline of insignificant p-values. Hierarchical clustering was performed by euclidean distance and average linkage. The individual plots show: (a) all genes represented with min. 2 spots; (b) all genes represented with min. 2 spots, both showing regulation in the same direction; (c) all genes represented with exactly 2 spots showing opposite regulation; (d) all genes represented with min. 3 spots and at least one up-and one down-regulated spot. For details see section ‘Differential Expression’ and ‘p-value combination’.

Figure 4 gives an overview over p-values obtained from the individual methods for the group comparison normal versus late (*n-l*). The dendrograms on the y-axes indicate the ‘relatedness’ of results. For all genes represented by at least 2 spots (sub-plot a), Stouffer’s method yields results that are most similar to the chi-square method and the variant thereof while p-values based on mean expression values are rather similar to the minimum of the underlying spot p-values. In the case of consistently regulated spots (b), these similarities become even more pronounced as can be deduced from the dendrogram branch lengths. For genes represented by exactly 2 spots which show opposite regulation (c), p-values from Stouffer’s method are most similar to the baseline (p-value = 1). For genes represented by at least 3 inconsistently regulated spots (d), Stouffer’s p-values are generally most similar to the baseline and the maximum spot p-value. These results indicate that Stouffer’s method returns conservative p-values for doubtful spot information yet it is as sensitive as e.g. the chi-square method for consistent spot information.

P-values derived from Stouffer’s method and mean expression values show high correlation for genes measured with more than one spot (Spearman’s rho 0.82). Lower correlation (0.53) was determined for genes represented by exactly two inconsistently regulated spots, for which Stouffer’s method produces in approx. 3/4 of the cases less significant p-values than those derived from combined expression values. For genes represented by multiple consistently regulated spots correlation is high (0.94), and Stouf-fer’s method produces in approx. 3/4 of the cases more significant p-values than those derived from combined expression values. Finally, for genes represented by multiple inconsistently regulated spots, p-values from both methods show moderate correlation (0.6), and no method shows clear tendency to return more significant p-values than the other. It is important to keep in mind that p-value combination make more significant p-values possible; genes represented by more spots/probe sets can achieve more significant p-values than those represented by fewer spots/probe sets. Thus, the number of spots for a given gene needs to be reported together with the p-value and fold-change. The argument that genes represented by few spots/probe sets are per se discriminated can be brought forward, yet on the other hand it appears reasonable that the detection of differential expression via multiple spots/probe sets increases overall confidence.

**Figure 5:**
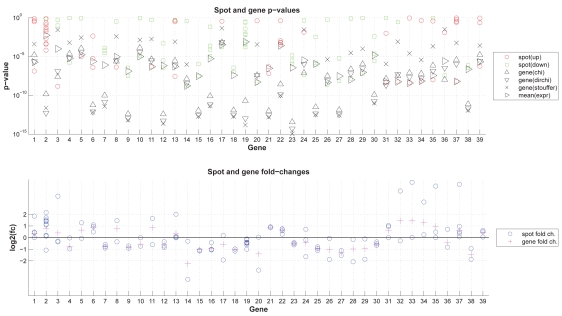
p-value combination—exemplary results: Different methods for combining spot p-values into gene p-values: Detailed results for selected genes. Upper figure: spot p-values and gene p-values derived from various methods; lower figure: spot and gene fold changes. For details see section ‘Differential Expression’ and ‘p-value combination’.

**Figure 6:**
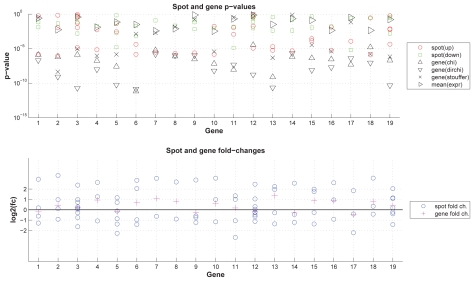
p-value combination—exemplary results for Affymetrix GeneChips: The plot shows, for exemplarily selected genes, p-values and fold-changes for probe sets and genes. The data was obtained from an U133 Plus 2.0 Affymetrix GeneChip. Upper figure: p-values for probe sets and genes; lower figure: fold changes for probe sets and genes. For details see section ‘Differential Expression’ and ‘p-value combination’.

The detailed results for exemplary genes (Fig. 5) illustrate the method’s behavior with regard to various numbers of individual spots with agreeing or disagreeing regulation direction. Genes 3, 8, 36 demonstrate Stouffer’s capability of penalizing disagreeing regulation direction; genes 2, 19, 22 show the important effect (i.e. more significant gene p-value) of combining individual evidence via p-value combination versus combining spot expression values. For Affymetrix GeneChips (Fig. 6) similar effects occur, i.e. in a number of cases probe sets representing a same gene show inconsistent regulation. Here, e.g. genes 7 and 8 were each measured by two probe sets showing opposite regulation with unequal probe set p-values, and both spots show similar intensity levels; for these genes Stouffer’s method returns p-values similar to those determined based on mean expression values. For genes with an unequal number number of up- and downregulated spots (e.g. genes 2, 4, 16), Stouffer’s p-values are generally more significant.

Overall, Fisher’s inverse chi-square method is most predominantly used for combining p-values among the analyzed methods. A drawback of this method is that it considers only absolute values of the p-values, i.e. the combination of two spots results in the same gene p-value irrespectively of their regulation direction, and the resulting gene p-value is of higher significance than the p-value of the corresponding spots.

The presented variant of Fisher’s inverse chi-square method partially eliminates this effect. If two spots have significant p-values and are regulated in opposite directions the resulting gene p-value is clearly less significant than the respective value of the original Fisher’s inverse chi-square method. Yet, p-values cannot cancel out each other; the more significant spot has higher influence and the less significant one has a minor, yet still increasing effect on the overall significance.

Stouffer’s method reflects p-values of opposite direction in a more prominent decrease in significance of the resulting overall p-value than the other methods for p-value combination. In Stouffer’s method, two spots of opposite directions and approximately equally significant p-values nearly cancel each other out. The comparison against combination of expression values (Figs. 4 and 5) indicates that Stouffer’s method generally returns very significant p-values for consistently regulated spots and conservative p-values for inconsistent spot information, thus it shows a high discriminative power. p-values based on the mean expression values do not depend on the number of spots representing a same gene, yet they are biased towards the spot p-value of the underlying spot with highest expression intensity. Despite its favorable properties, Stouffer’s method has so far been, to our knowledge, predominately been used in studies aiming at integration of various types or sets of previous results (meta-analysis, e.g. for integration of medical studies [43,44,45] and in social sciences [46]), we are not aware of its application for combining spot p-values into gene p-values. Besides the widely used methods analyzed here, a number of other more rarely used methods for combining p-values exist (e.g. [22,21]).

The analyzed methods for p-value combination assume statistical independence of the input data. Spot p-values are not necessarily independent. Microarray studies often entail experimental validation which generally is labor-intensive and, thus, only a certain number of genes can be further investigated. Therefore, a rather limited number of top p-value candidates is of interest, often pre-filtered for significant fold-change; these genes are required to be differentially expressed with high confidence. Gene p-values are predominently used for ranking genes and for giving a rough estimate of statistical significance; the p-values derived from Stouffer’s method are perfectly suited for these tasks, especially as they reflect consistent and inconsistent regulation in a more meaningful manner than other methods.

This combination method was applied in all other presented analyses based on gene p-values if not indicated otherwise.

### Number of regulated genes

**Figure 7:**
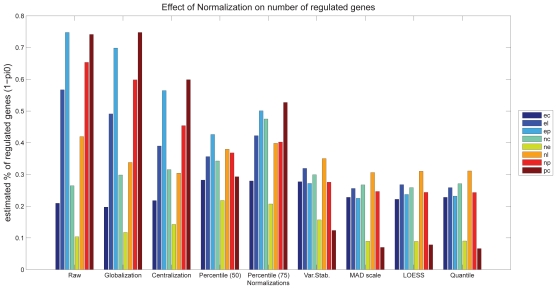
Number of significantly regulated genes: Effect of normalization on the number of significantly regulated genes, which is estimated from the distribution of p-values.

Figure 7 shows that the estimated number of significantly regulated genes varies significantly for different group comparisons and different normalizations. The effect of normalization is most pronounced for the comparison *pc*; depending on the normalization method between 7% and 74% of the genes appear regulated. The expectation is to find less genes to be regulated in the comparisons *ne* and *pc* compared to *np* and *nc*. Only a subset of the analyzed normalization methods yield results which support this expectation, these are the percentile normalization to the median, Variance Stabilization, MAD scale normalization, LOESS and Quantile normalization. The latter four yield significantly smaller numbers of regulated genes in all comparisons than other methods.

The estimation of the number of differentially expressed genes via the R-library ‘qvalue’ is straight forward to apply. We propose this method for quality control of normalizations, especially for multi-group comparisons. As this approach returns a single number per group comparison and normalization, the overall number of regulated genes and the specific pattern of various group comparisons can easily be checked against background knowledge. This method thus represents a very useful method for testing the appropriateness of normalization methods.

### Robustness analysis

**Figure 8:**
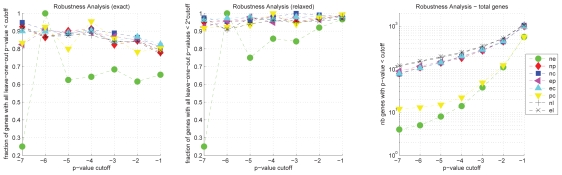
Robustness Analysis of p-value calculation: leave-one-out analysis. Fraction of genes significant at a certain p-value level in the overall p-value calculation that are also significant in the leave-one-out p-value calculation according to two different measures for evaluation *exact* (left panel) and *relaxed* (middle). The right panel shows the total number of genes significant at a certain p-value-level for each group comparison. The plots shown here were obtained with MAD-normalized data. For details see section ‘Robustness analysis’.

**Figure 9:**
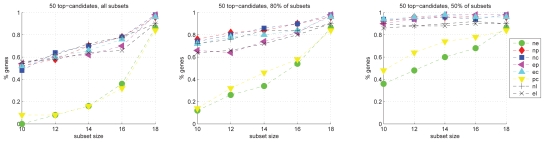
Robustness Analysis of p-value calculation: Subset sampling. Fraction of the 50 top p-value candidates in the overall p-value calculation that are also among the 50 top candidates in at least *s*% of the subset-based p-values. Left: all subset p-values (*s* = 100); middle: *s* = 80; right: *s* = 50. The plots shown here were obtained with MAD-normalized data. For details see section ‘Robustness analysis’.

The large number of samples available in the OA-dataset allows us to assess the robustness of the differentially regulated genes between two sample groups. The exemplary results shown in Figures 8 and 9 were obtained with MAD-scale normalized data and Stouffer’s method for differentially regulated genes.

#### Leave-one-out analysis

The results of this analysis (Fig. 8) show that the p-values are generally very robust. Considering a cutoff p-value of 10^−3^ the agreement of most group comparisons covers >82% of all genes in the strict analysis and >93% in the relaxed analysis. The comparison of p-values between normal and early degenerative cartilage shows the smallest robustness; one reason for this is the small number of significantly regulated genes in this comparison (only 8 genes have a p-value = 10^−5^, 38 genes have a p-value = 10^−3^), and this also reflects the relatively high similarity of normal and early degenerative cartilage samples.

Overall, this confirms that the applied methods for normalization and p-value combination yields robust p-values and, thus, the genes selected on the basis of these p-values or the corresponding q-values can be assumed to be appropriate for further biological investigation. Our analysis shows that an error of about 10% of the significantly differentially regulated genes has to be expected.

#### Subset sampling

The result of subset sampling for *t* = 50 (*t* = 75 and *t* = 100 yield very similar results) is shown in Figure 9. The figure shows that the p-values are of varying stability. Generally, the fraction of stable genes increases when the number of samples in the subset (*m*) increases. The genes for the group comparisons *ne* and *pc* are significantly less robust than the other comparisons. The other group comparisons show higher stability; for a subset sample size of 10, about 50% of the top-candidates are present in all subset p-value top-candidates; about 90% of the top-candidates are present in half of the subset top-candidates. For these group comparisons, the fraction of stable genes also rises with increasing subset size, but this increase is rather modest compared to *ne* and *pc*. In any case, the analysis yields an overview of the error and variance to be expected within the respective group comparison and the involved differentially regulated genes.

Robustness analysis has previously been used as a means for estimating the stability of the top-candidates derived from group comparisons (e.g. [23]). We propose it as a means for testing biological hypotheses and appropriateness of normalization. For the given data set, we know that degree of similarity between the sample groups varies substantially. This is reflected in p-value robustness; for appropriately normalized data the top-candidates of comparisons of clearly distinct groups are more robust than top-candidates of very similar groups.

## Conclusions

Our study shows that microarray data normalization and processing have important effects on the final outcome, especially for the identification of differentially expressed genes. Generally, a normalization method should modify the original data as little as possible. Methods that modify data more importantly can become necessary if the data shows abnormal effects that cannot be covered by moderate normalization methods. Prior biological knowledge is helpful when deciding on the appropriateness of a method under investigation. It is evident that prior biological knowledge is not always available and thus no requirements with respect to the expression levels and/or differentially expressed genes can be formulated, e.g. if so far unknown biological phenomena are studied with small-scale microarrays. In this case, generally well performing methods should be applied and, if possible, results should be validated by additional experiments (e.g. RT-PCR).

We propose the following guidelines and procedures that should be performed after any normalization to test its appropriateness. All these analyses produce quality measures which need to be inspected and checked against prior biological knowledge. The corresponding check list can be formulated as follows:

1. Does the data show systematic differences in expression levels for the different sample groups, and are these potential effects of the biological phenomena under investigation? ⇒ inspection of group-level plots.
2. Is the distribution of fold-change and p-value compatible with prior biological knowledge? ⇒ analysis of shape of volcano-plots (e.g. symmetry if appropriate).
3. Does the estimated number of differentially regulated genes agree with biological expectation? Generally, for most experiments only a small fraction of genes is expected to be regulated. In the case of multi-group comparisons, does the pattern of these numbers for the set of comparisons contradict prior knowledge? ⇒ estimation of the number of differentially expressed genes.
4. Are the most significantly differentially expressed genes consistently significant when applying subset sampling? For multi-group comparisons, does the pattern of top-candidate stability correspond to prior knowledge? ⇒ leave-one-out and subset-sampling analysis for estimating the reliability of the genes detected as being differentially expressed.

Furthermore, we analyzed methods for integrating spot data into gene data, an important task that has been somewhat neglected so far. We showed that p-value combination has some important advantages over combination of expression values in terms of sensitivity, robustness against opposite regulation direction and independence of varying expression intensities for individual spots. We found Stouffer’s method for combining p-values to correspond best to the imposed requirements. According to our experience, Stouffer’s method is the only available method yielding plausible results if genes are resampled by multiple spots on a chip and if these might exhibit inconsistent fold-changes and significantly varying expression levels. For the given data sets, Affymetrix did not show as prominent variation in expression levels as the cDNA data set, yet the method represents a useful control in absence of a gold standard and a valuable alternative to the standard method based on mean expression levels. This method has not been described before for this task.

In summary, this study shows on exemplary data that it is of vital importance to check every individual step of gene expression data analysis for its appropriateness. Certainly, gene expression data analysis has to fit statistical requirements, but it also needs to account for experimental and biological background knowledge. For most individual processing steps numerous alternatives exist and, therefore, it is important to test different possibilities and analyze the effects of the decision with appropriate tools and measures. For large enough measurements (approx. 20 samples per group) the use of global robustness and quality measures, obtained e.g. via the subset sampling approach, can help in estimating the reliability of final microarray study results.
